# Distinct Outcomes of Oropharyngeal Squamous Cell Carcinoma Patients after Distant Failure According to p16 Status: Implication in Therapeutic Options

**DOI:** 10.3390/curroncol28030156

**Published:** 2021-04-29

**Authors:** Anouchka Modesto, Aurore Siegfried, Amelie Lusque, Sébastien Vergez, Jerome Sarini, Laurent Brouchet, Emmanuelle Uro-Coste, Pierre Graff-Cailleaud, Jean Pierre Delord

**Affiliations:** 1 Radiation Oncology Department, Institut Claudius Regaud, Institut Universitaire du Cancer de Toulouse, CEDEX 09, 31059 Toulouse, France; 2 Pathology Department, Centre Hospitalo-Universitaire de Toulouse, Institut Universitaire du Cancer de Toulouse, CEDEX 09, 31059 Toulouse, France; siegfried.a@chu-toulouse.fr (A.S.); uro-coste.e@chu-toulouse.fr (E.U.-C.); 3 Biostatistics Department, Centre Hospitalo-Universitaire de Toulouse, Institut Universitaire du Cancer de Toulouse, CEDEX 09, 31059 Toulouse, France; lusque.amelie@iuct-oncopole.fr; 4 Head and Neck Surgery Department, Centre Hospitalo-Universitaire de Larrey, Institut Universitaire du Cancer de Toulouse, CEDEX 09, 31059 Toulouse, France; vergez.sebastien@iuct-oncopole.fr (S.V.); sarini.jerome@iuct-oncopole.fr (J.S.); 5 Thoracic Surgery Department, Centre Hospitalo-Universitaire de Larrey, CEDEX 09, 31059 Toulouse, France; brouchet.l@chu-toulouse.fr; 6 Radiation Oncology Department, Institut Curie, CEDEX 05, 75005 Paris, France; pierre.graff@curie.fr; 7 Medical Oncology Department, Institut Claudius Regaud, Institut Universitaire du Cancer de Toulouse, CEDEX 09, 31059 Toulouse, France; Delord.Jean-Pierre@iuct-oncopole.fr

**Keywords:** oligometastases, human papillomavirus, stereotactic ablative radiotherapy, lung metastases, oropharyngeal squamous cell carcinoma

## Abstract

Introduction: Recent modifications in the epidemiology of oropharyngeal squamous cell carcinoma (OSCC) have led to the increase of Human papillomavirus (HPV) related metastatic head and neck cancer patients with high life expectancy even at advanced stage, low comorbidity and still restricted systemic therapy opportunities. In the recent era of ablative therapies’ development, oligometastatic HPV OSCC patients are indubitably good candidates for intensified treatment. However, data related to outcomes after optimised management of metastatic sites are dramatically missing. Materials and patients: In our cohort of 186 unselected consecutive OSCC patients treated with curative intent at our institution between 2009 and 2013, we analysed the incidence, treatment and outcomes of distant metastatic (DM) failure according to p16 status. Results: After a median follow-up of 4.2 years (95% CI: 3.8–4.4) from primary diagnosis of OSCC, 21/95 p16− patients (22.1%) vs. 8/91 (8.8%) p16+ patients presented DM failure with a median interval of 11 (range 0–46) and 28 months (range 0–71), respectively (*p* = 0.10). Overall survival (OS) after DM failure was significantly higher in p16+ patients with a two-year OS rate of 75% and 15% for p16+ and p16−, respectively (*p* = 0.002). In eight HPV-related metastatic patients, three underwent ablative lung metastasis treatment and are still complete responders four to five years later. Conclusion: This study highlights distinct outcomes of metastatic HPV-related OSCC patients emphasised by three long-term complete responders after lung ablative treatment. In patients with high life expectancy and limited tumour burden, the question of ablative treatment such as metastasectomy or stereotactic ablative radiotherapy (SBRT) should be addressed.

## 1. Introduction

Over the past few decades, the epidemiology of oropharyngeal squamous cell carcinoma (OSCC) witnessed an increased proportion of cases related to the infection with oncogenic subtypes Human Papillomavirus (HPV) 16 or 18, reaching 50% to 70% in some countries [1,2]. HPV-related (HPV+) OSCC occur in healthier individuals with little or no history of tobacco consumption. Despite a high rate of nodal extension at diagnosis, their prognosis is strikingly better than HPV-unrelated OSCC patients even at metastatic stage [3]. This has led to the validation of a specific prognostic staging system for OSCC based on tumour p16 status, which is considered as a reliable surrogate marker for HPV-driven carcinogenesis [4].

Historically, metastatic head and neck cohorts included mostly HPV-unrelated SCC and were associated with a tragic prognosis [5]. The current first-line standard systemic treatment for metastatic SCC patients combines platinum-based chemotherapy regardless of p16 status [6,7]. When the number of metastases is limited (so-called oligometastatic disease), local ablative therapies can be proposed to target every single metastasis. In the recent era of ablative therapies, oligometastatic patients of various histologies benefit greatly from local treatment compared to standard systemic regimens [8,9,10].

In a large cohort of consecutive OSCC patients treated with curative intent in our institution, we reviewed the incidence, management and outcome of distant metastatic (DM) failure according to the primary tumour’s p16 status with a focus on the outcome of oligometastatic p16+ patients managed by ablative therapy of their pulmonary metastases.

## 2. Materials and Methods

As part of an institutional board-approved study, all consecutive patients treated with curative intent at our tertiary cancer centre for an OSCC between January 2009 and December 2013 were identified (*n* = 278). Clinical records were retrospectively reviewed to verify patients and tumour characteristics, treatment details and clinical outcomes. Ninety-two patients were excluded for the following reasons: multiple synchronous tumour sites (*n* = 22); history of prior head and neck carcinoma (*n* = 20); 3-dimensional conformal radiotherapy (*n* = 26); unavailable material for p16 reviewing (*n* = 24).

Of 186 OSCC patients, 91 (49%) were p16+. Treatment modalities of the primary tumour have been previously described [11]. Briefly, en bloc oropharyngectomy with uni- or bilateral neck dissection was performed when feasible followed by adjuvant radiotherapy in the event of T3/T4 stage, close margins (<5 mm) or nodal involvement. Concurrent chemotherapy (cisplatin) was delivered in the event of clear final margins ≤ 1 mm or extracapsular spread. Alternatively, patients were treated by definitive radiotherapy with concurrent cisplatin or cetuximab. The prescribed doses were 70 and 56 Gray delivered in 35 fractions using intensity-modulated radiotherapy (IMRT) with simultaneous integrated boost to the high- and low-risk volumes, respectively.

Follow-up included a physical examination by direct fibre-optic nasopharyngeal-laryngoscopy performed by a radiation oncologist or a head-and-neck surgeon every 3 months for 2 years, then every 6 months for 3 years. Contrast-enhanced CT evaluation was performed 3 months after treatment completion and annually thereafter, or if failure was suspected. Each case of DM failure was discussed by our head-and-neck tumour board. In the event oligometastatic disease, local ablative treatment (stereotactic body radiotherapy or surgery) was validated by head and neck dedicated tumour board.

Immunostaining of p16INK4A was reviewed in all 186 patients on 3-µm-thick formaldehyde fixed, paraffin-embedded tissue sections, which were deparaffinised using high pH solution. As a primary p16INK4A antibody, clone E6H4 (mouse monoclonal, 1:30 dilution, Roche, Almere, The Netherlands) was used and detected using Powervision (DAKO A/S, Glostrup, Denmark) and peroxidase-DAB visualisation [4]. P16 overexpression (p16+) was assessed by p16INK4A as continuous strong nuclear staining with or without cytoplasmic staining observed in all tumour cells. In each analysis, negative and positive controls were assessed.

### Statistical Analysis

Descriptive statistics were presented as median (range) for continuous variables and numbers (%) for categorical variables. Comparisons between groups were assessed using the Kruskal–Wallis test for continuous variables and the Chi-squared or Fisher exact test for categorical variables. OS after DM failure was defined as time from metastatic diagnosis to death. Time-to-event outcome was estimated using the Kaplan–Meier method and compared using the log-rank test. All tests were two-sided and a *p*-value < 0.05 was considered significant. All statistical analyses were conducted by using STATA version 13.

## 3. Results

**Figure 1:**
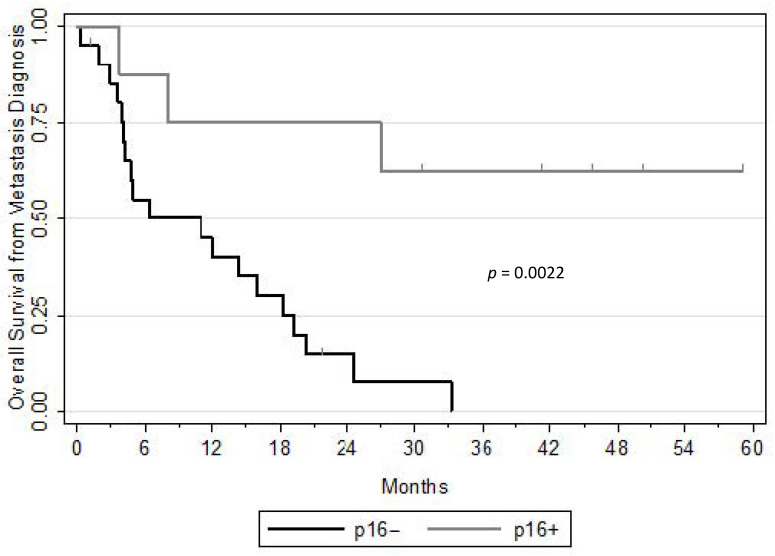
Overall survival after distant failure according to p16 status *n* = 29.

With a median follow-up from the primary diagnosis of 4.2 years (95%CI: 3.8–4.4), 21/95 (22.1%) p16− patients versus 8/91 (8.8%) p16+ patients developed a DM. The median interval between the primary diagnosis and the occurrence of DM failure was 11 (range 0–46) and 28 months (range 0–71), for p16− and p16+ patients, respectively (*p* = 0.10). Table 1 shows patients’ characteristics and primary treatment modalities. At metastatic recurrence, all patients received standard first line systemic therapy or best supportive care, except three HPV+ patients who underwent only local ablative treatment of lung metastases. With a median follow-up from the diagnosis of distant relapse of 3.8 years, the 2-year OS rate was 15% and 75% for p16− and p16+ patients, respectively (*p* = 0.0022, Figure 1).

The three p16+ patients treated only with a local ablative treatment of an isolated lung metastasis are alive and disease-free four to five years after salvage treatment. Patient 1: This 75-year-old man with no history of alcohol or tobacco consumption was diagnosed in 2011 with a p16+ squamous cell carcinoma of the base of tongue with ipsilateral lymph nodes (Supplementary data Figure S1A). He underwent right oropharyngectomy with ipsilateral nodal dissection and free flap reconstruction. Final resected margins were >3 mm and the three involved lymph nodes did not present extracapsular spreading (AJCC 7th edition T2N2b). Adjuvant RT delivered 54 Gy in 27 fractions to the tumour bed and the bilateral neck. During follow-up, he developed an isolated 18F-FDG avid pulmonary nodule in the left superior lobe increasing from 4 mm to 9 mm from April to July 2014 (Supplementary data Figure S1B). Despite noncontributory CT-guided biopsy, the nodule was considered as a lung metastasis of the head-and-neck tumour and treated with stereotactic ablative body radiotherapy (SBRT) in September 2014 (55 Gy in 5 fractions, Supplementary data Figure S1C). With more than five years of follow-up, the patient is still a complete responder on the last 18F-FDG TEP in September 2019 (Supplementary data Figure S1D).

Patient 2: This 79-year-old man with no history of alcohol or tobacco consumption was diagnosed in 2013 with a left tonsillar p16+ squamous cell carcinoma with ipsilateral nodal extension. He underwent an inaugural left nodal dissection. Eight of 24 lymph nodes removed were involved (15 to 30 mm) with six presenting extracapsular spreading (AJCC T2N2b). Definitive IMRT was delivered to the unresected primary tumour with bilateral prophylactic neck irradiation (70, 63 and 54 Gy in 35 fractions) with concurrent cisplatin. In September 2015 he developed an isolated 18F-FDG avid pulmonary nodule in the left superior lobe considered as a lung metastasis and treated with SBRT in October 2015 (55 Gy in 5 fractions, Figure S2A). Subsequently, he developed another isolated nodule in the paramediastinal left lung (17 mm) that was resected in April 2016 (Figure S2B). The final pathology report revealed squamous cell carcinoma morphologically similar to the primary tumour with intense p16 staining. The specimen was positively stained by chromogenic in situ hybridization (CISH) with Automate Ventana Benchmark ULTRA high risk HPV III Family 16 (Supplementary data Figure S2C).

Resection was complete. The patient was disease-free on the last 18F-FDG PET scan in January 2020 (Supplementary data Figure S2D).

Patient 3. This 71-year-old woman, a former smoker (40 pack-years) but with no history of alcohol consumption, was diagnosed in December 2013 with a p16+ squamous cell carcinoma of the tongue base. There was a synchronous single 45-mm pulmonary nodule in the right inferior lobe but no evidence of nodal extension (T2 N0). Biopsies from both sites revealed p16+ positivity for squamous cell carcinoma (Figure S3A). She initially underwent right inferior lobectomy associated with mediastino-hilar dissection (Supplementary data Figure S3A). The final pathology report revealed squamous cell carcinoma with intense p16 staining (Supplementary data Figure S3B). The specimen was positively stained by chromogenic in situ hybridization (CISH) with Automate Ventana Benchmark ULTRA high risk HPV III Family 16. Resection was complete and there was no evidence of hilar or mediastinal nodal extension. In June 2014 she received definitive radiotherapy to the primary tumour with bilateral prophylactic neck irradiation of 66 Gy and 54 Gy in 30 fractions (Supplementary data Figure S3C) with concurrent weekly cetuximab (6 cycles). She was disease-free on the last CT scan in November 2018 (Supplementary data Figure S3D).

## 4. Discussion

This study emphasizes the distinct outcome of HPV+ OSCC patients compared to those with HPV-unrelated OSCC at metastatic stage. We confirm previous reports that patients with HPV+ disease present delayed DM failure (28 vs. 11 months (*p* = 0.10)) and have better OS after DM failure than those with HPV-unrelated OSCC: the two-year OS rate was 75% and 15%, respectively (*p* = 0.0022) [12]. Moreover, three patients presented oligo-metastatic lung failure eligible for ablative treatment (surgical removal or SBRT) without any evidence of failure more than four to five years of follow-up from diagnosis. To our knowledge, only two articles reported a complete response after optimal local treatment for distant metastatic sites in oligometastatic HPV-related OSCC patients: a case with a synchronous single brain metastasis with a complete response six months after whole brain RT completion, and a series of four maintained complete responders after metachronous lung metastases resection (follow-up 0.9–3.4 years) [13,14]. HPV+ OSCC are often diagnosed with nodal extension and are associated with non-negligible risk of distant metastases [15]. Even at metastatic stage, striking differences have been observed between the prognoses of HPV+ OSCC patients and HPV-unrelated ones [16,17]. Distant metastases generally occur significantly later (beyond two years) and with a disseminating phenotype (more than two organs) in a non-negligible proportion of patients [18]. HPV-positive OPSCC has improved survival in the setting of distant metastatic presentation as compared with HPV-negative disease and shows greater responsiveness to treatment [19]. Oligometastatic status has been previously reported to be associated with improved survival in this population [20]. Thus far, there is no prospective data supporting a distinct systemic management from other head and neck patients except in the context of a clinical trial [21]. The management of metastatic solid tumours has historically focused on systemic treatment given with palliative intent. The spectrum hypothesis of cancer postulates that metastatic disease exists in a continuum ranging from loco-regional cancer to widespread metastases [22]. At present, the oligometastatic stage refers to 1 to 5 sites of radiographically detected macroscopic metastases [23]. Their low tumour burden and the limited effect of standard systemic therapy led to the concept of iterative optimised local management to delay the introduction of chemotherapy [24]. The concept of resecting metastatic disease with curative intent is counterintuitive to the concept that metastases represent a systemic disease. However, if one envisions the metastatic disease to be regionally confined to a specific organ then acceptance of an optimised local therapy such as SBRT or surgery of an optimised local therapy is reasonable [25]. In some histologies associated with prolonged survival at the metastatic stage such as colorectal cancer, prostate or breast carcinoma, extensive local management of the metastatic sites with SBRT in case of limited tumour burden was correlated with tumour control and postponing systemic treatment [26]. In recent decades, the resection of colorectal liver metastases has become a standard of care leading to improved outcomes and sometimes a cure in well-selected patients [27]. The recent pooled analysis of 700 patients with medically inoperable lung metastases of various histologies treated with SBRT reported a local control rate of 81.2% with 6.5% of ≥grade 2 pneumonitis [28]. Considering their high life expectancy, oligometastatic HPV+ patients are indubitably good candidates for optimised local treatment to delay the introduction of standard chemotherapy and obtain complete response in well- selected patients that remain to be further elucidated.

Our study suffers from several limitations besides its restricted number of cases and its retrospective nature. One of the major issues of isolated lung extension diagnosed during the management or the follow-up of head and neck patients is represented by the impossibility to distinguish a metastatic location from a distinct primary squamous cell carcinoma. This is due to extensive morphologic overlap, especially when there is a history of tobacco consumption. It can be partly overcome by the overexpression of p16, a cell cycle regulator protein whose positivity in immunochemistry is considered as a reliable surrogate marker of HPV status in oropharyngeal carcinoma [29]. Another issue is the lack of histological proof in one patient. However, radiographic evidence such as peripheral sites, lack of spiculation and the absence of nodal lung or mediastinal extension can be helpful tools to determine whether an isolated nodule is a primary or a secondary disease [30].

## 5. Conclusions

In conclusion, this study underlines the distinct outcomes of HPV-related OSCC patients compared to HPV-unrelated patients at metastatic stage emphasised by three long-term complete responders after lung ablative treatment. This highlights the need for prolonged follow-up for these patients in order to detect and treat relapse at an early stage. In cases of high life expectancy and limited tumour burden, the question of ablative treatment such as metastasectomy or SBRT should be addressed.

## Figures and Tables

**Table 1:** Demographics, disease and treatment characteristics of metastatic patients (*n* = 29) and according to p16 status.

Characteristics	Overall Cohort (*N* = 29)	p16− (*N* = 21)	p16+ (*N* = 8)	*p*-Value
Age, median (range)	55 (42–79)	55 (42–79)	55 (49–73)	0.23
Gender M/F (%)	21 (72)/8 (28)	15 (71)/6 (29)	6 (75)/2 (25)	1.00
Performance status 0/1–3 (%)	11 (38)/18 (62)	5 (24)/16 (76)	6 (75)/2 (25)	0.028
Tobacco consumption > 10 pack-year (%)	21 (81) 3 missing	18 (100) 3 missing	3 (38)	0.001
Alcohol abuse (%)	20 (69)	18 (86)	2 (25)	0.004
Primary tumour stage T1–2/T3–4 (%)	11 (38)/18 (62)	6 (29)/15 (71)	5 (62.5)/3 (37.5)	0.20
Primary nodal stage *N*0–*N*1/*N*2–*N*3 (%)	12 (41)/17 (59)	10 (48)/11 (52)	2 (25)/6 (75)	0.41
Lung synchronous metastasis (%)	2 (7)	1 (5)	1 (12.5)	
Locations (%) -Tonsil -Glossotonsillar sulcus -Base of tongue	14 (48) 12 (42) 3 (10)	10 (48) 8 (38) 3 (14)	4 (50) 4 (50) 0 (0)	
-Initial primary treatment (%) -Definitive RCT -Surgery +/− adjuvant RCT -Induction chemotherapy (%) -Concurrent systemic therapy (%) -Cisplatin (%), *n* = 14 -Cetuximab (%), *n* = 7	19 (65.5) 10 (34.5) 7 (24) 21 (72) 14 (67) 7 (33)	15 (71) 6 (29) 7 (33) 16 (76) 9 (56) 7 (44)	4 (50) 4 (50) 0 (0) 5 (62) 5 (100) 0 (0)	

## Data Availability

Data are unavailable due to patient’s confidentiality.

## Supplementary Materials

The following are available online at https://www.mdpi.com/article/10.3390/curroncol28030156/s1, Figure S1: Patient 1, Figure S2: Patient 2, Figure S3: Patient 3.
