# Supplementary material for: Distinct Outcomes of Oropharyngeal Squamous Cell Carcinoma Patients after Distant Failure According to p16 Status: Implication in Therapeutic Options
Source: Curr Oncol. 2021 Apr 29;28(3):1673–80. doi: 10.3390/curroncol28030156 (PMC8161744; doi:10.3390/curroncol28030156)
Supplement: Supplementary file 1 [file curroncol-28-00156-s001.zip › curroncol-1176042-supplementary.pdf]

Figure S1. Patient 1

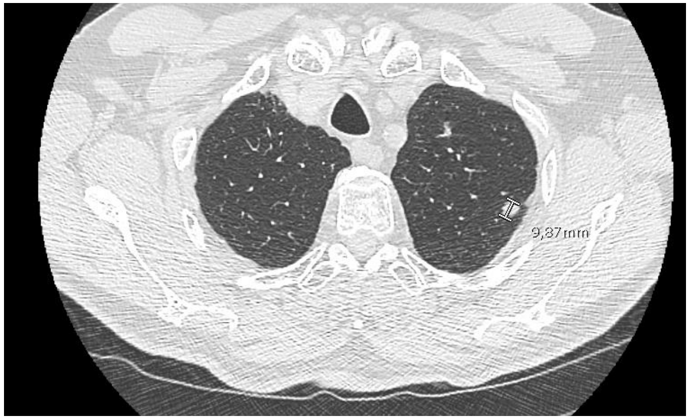

1.A TDM June 2014

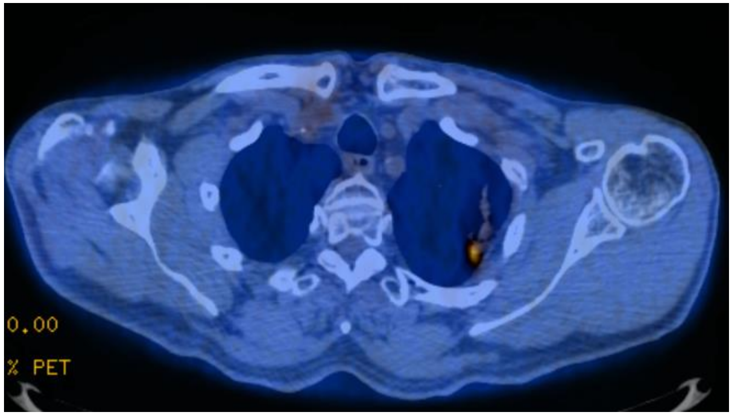

1.B FDG TEP July 2014

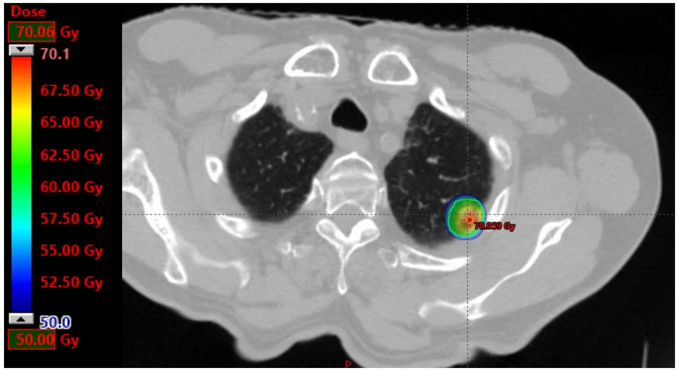

1.C SBRT planification 55 Gy in 5 fractions September 2014

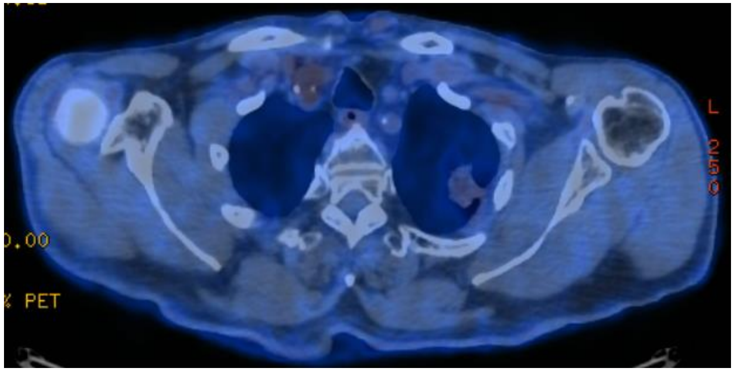

1.D FDG TEP September 2019

Figure S2. Patient 2

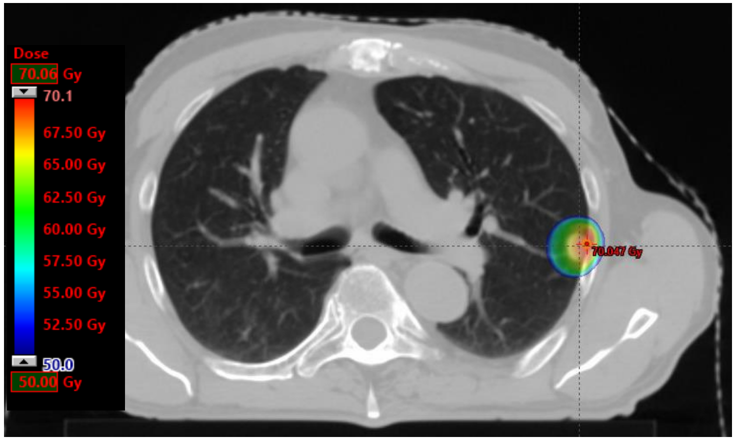

2.A SBRT planification 55 Gy in 5 fractions October 2015

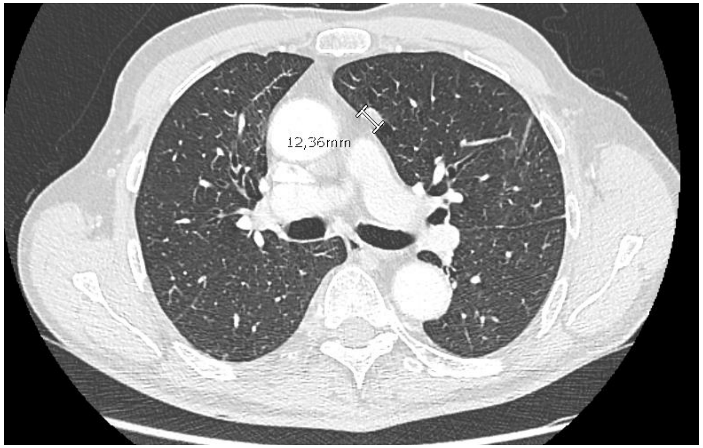

2.B para mediastinal metastasis September 2016

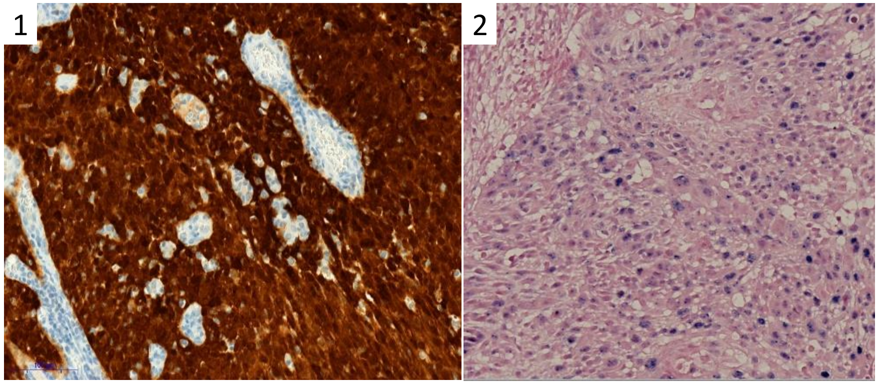

2.C Mediastinal resection November 2016 : p16 IHC overexpression (1) and HPV high risk overexpression CISH (2)

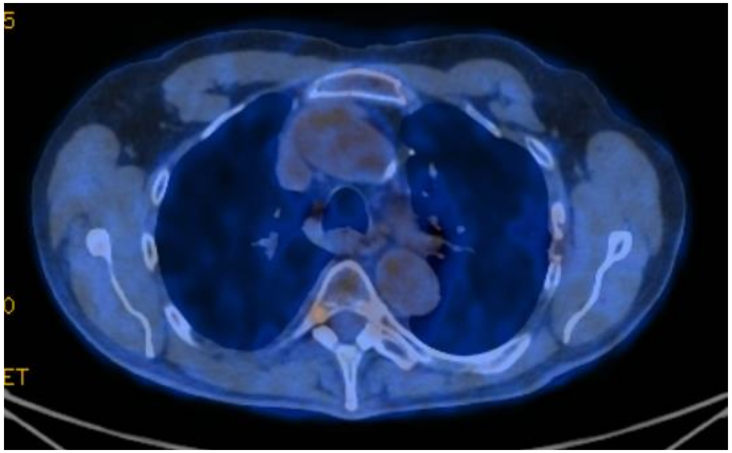

2.D FDG TEP January 2020

Figure S3. Patient 3

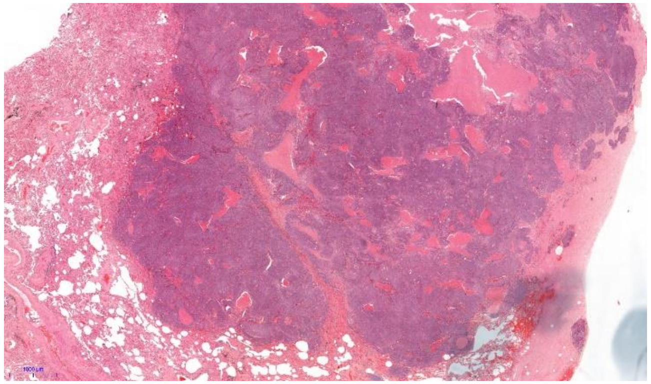

3.A Hematoxylin Eosin of one right inferior lobar metastase December 2013

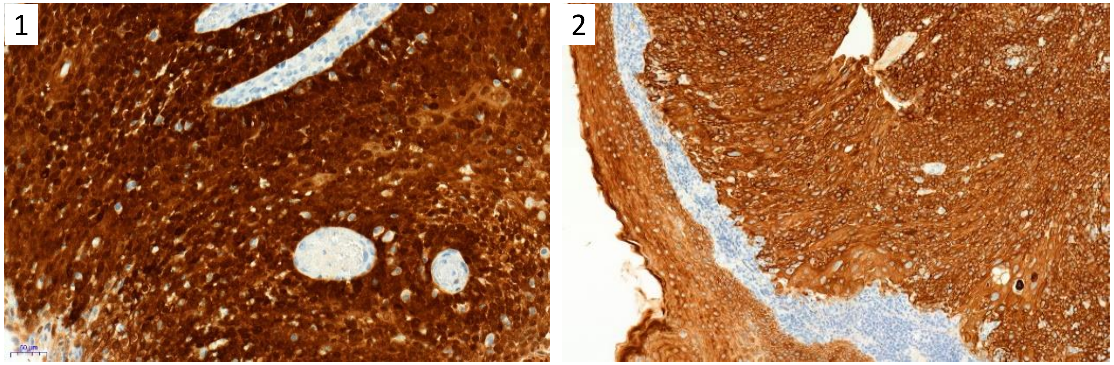

3.B January 2014 lung (1) and oropharyngeal (2) p16 IHC overexpression

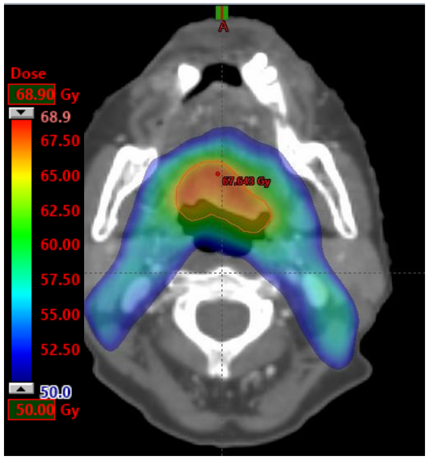

3.C OSCC RT planification 66 Gy in 30 fractions April 2014

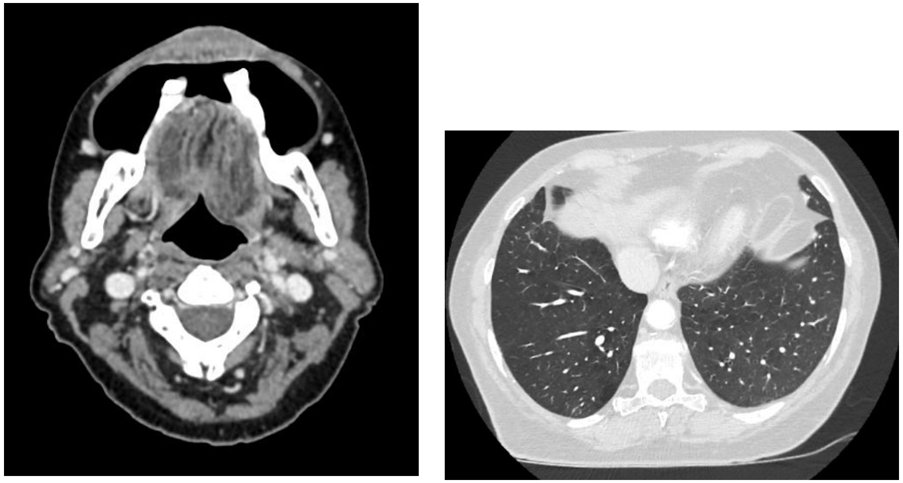

3.D Complete response November 2018
